# (*Z*)-6-{2-[(*E*)-2,4-Dihydroxy­benzyl­ideneamino]phenyl­amino­methyl­ene}-3-hydroxy­cyclo­hexa-2,4-dienone toluene solvate

**DOI:** 10.1107/S1600536808026305

**Published:** 2008-08-20

**Authors:** Hoong-Kun Fun, Reza Kia, Valiollah Mirkhani, Hasan Zargoshi

**Affiliations:** a X-ray Crystallography Unit, School of Physics, Universiti Sains Malaysia, 11800 USM, Penang, Malaysia; b Chemistry Department, University of Isfahan, Isfahan 81746-73441, Iran

## Abstract

The bis-Schiff base title compound, C_20_H_16_N_2_O_4_·C_7_H_8_, crystallized as a toluene solvate. In the solid state, it is present as its prototropic tautomer formed by transfer of one of the *ortho*-hydroxyl H atoms. The proton transfer is accompanied by a shift of electron pairs, as is evident from the observed C—O and C—N bond distances of 1.305 (2) and 1.315 (2) Å, which are largely consistent with C=O and C—N distances. The actual mol­ecule present in the solid state is thus the charge-neutral β-keto amine, with a small contribution of its zwitterionic valence tautomer *via* partial delocalization of electron pairs along the N—C—C—C—O atom chain. The dihedral angles between the central benzene ring and the two outer benzene rings of the Schiff base are 51.99 (8) and 12.95 (9)°. Intra­molecular O—H⋯N and N—H⋯O hydrogen bonds generate *S*(6) ring motifs, whereas intra­molecular N—H⋯N hydrogen bonds generate *S*(5) ring motifs. In the crystal structure, O—H⋯O hydrogen bonds and weak C—H⋯O inter­actions link the mol­ecules into one-dimensional zigzag chains along the *b* axis; these chains are further stacked by O—H⋯O and weak C—H⋯O inter­actions along the *c* axis, forming two-dimensional extended networks parallel to the *bc* plane. In addition, the crystal structure is further stabilized by weak C—H⋯π and π–π inter­actions.

## Related literature

For bond-length data, see: Allen *et al.* (1987 ▶). For details of hydrogen-bond motifs, see: Bernstein *et al.* (1995 ▶). For related structures, see, for example: Cakir *et al.* (2002 ▶); Eltayeb *et al.* (2007*a* ▶,*b* ▶); Karabiyik *et al.* (2007 ▶); Fun, Kargar & Kia (2008 ▶); Fun, Kia & Kargar (2008 ▶); Fun, Mirkhani *et al.* (2008**a* ▶,b* ▶). For background on applications of Schiff base ligands, see, for example: Hajioudis *et al.* (1987 ▶); Granovski *et al.* (1993 ▶); Dao *et al.* (2000 ▶); Shahrokhian *et al.* (2000 ▶); Eltayeb & Ahmed (2005**a* ▶,b* ▶); Fakhari *et al.* (2005 ▶); Karthikeyan *et al.* (2006 ▶); Sriram *et al.* (2006 ▶). For related literature, see: Fun & Kia (2008 ▶).
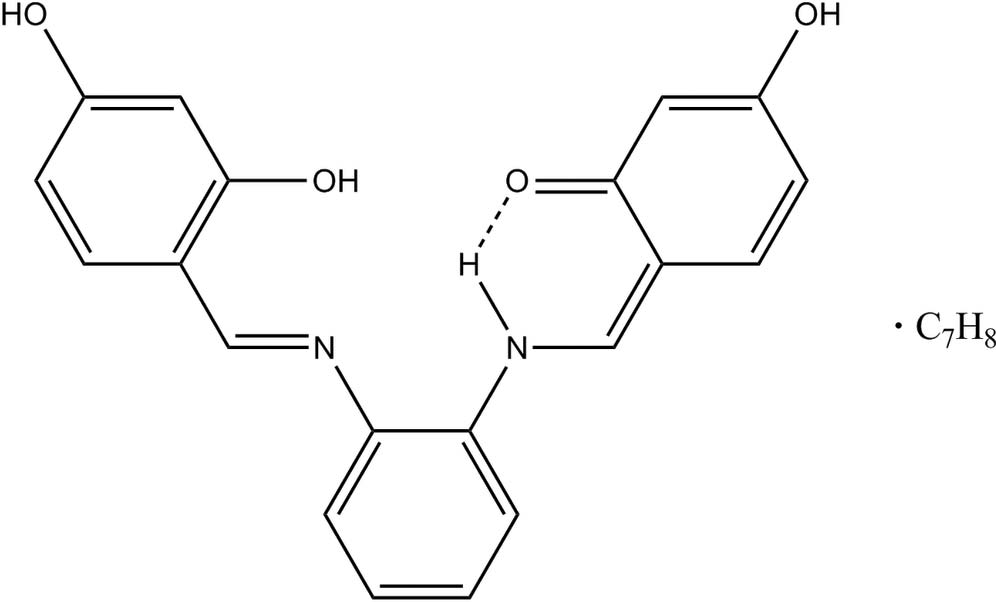


## Experimental

### 

#### Crystal data

C_20_H_16_N_2_O_4_·C_7_H_8_
*M* *_r_* = 440.48
Monoclinic, 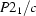

*a* = 11.9753 (3) Å
*b* = 18.8539 (5) Å
*c* = 9.9240 (2) Å
β = 108.819 (1)°
*V* = 2120.87 (9) Å^3^
*Z* = 4
Mo *K*α radiation
μ = 0.09 mm^−1^
*T* = 100.0 (1) K
0.25 × 0.13 × 0.02 mm

#### Data collection

Bruker SMART APEXII CCD area-detector diffractometer
Absorption correction: multi-scan (**SADABS**; Bruker, 2005 ▶) *T*_min_ = 0.954, *T*_max_ = 0.994
24830 measured reflections
6233 independent reflections
4023 reflections with *I* > 2σ(*I*)
*R*_int_ = 0.038

#### Refinement

*R*[*F* ^2^ > 2σ(*F* ^2^)] = 0.058
*wR*(*F* ^2^) = 0.163
*S* = 1.11
6233 reflections
299 parameters
H-atom parameters constrained
Δρ_max_ = 0.76 e Å^−3^
Δρ_min_ = −0.32 e Å^−3^

### 

Data collection: *APEX2* (Bruker, 2005 ▶); cell refinement: *APEX2*; data reduction: *SAINT* (Bruker, 2005 ▶); program(s) used to solve structure: *SHELXTL* (Sheldrick, 2008 ▶); program(s) used to refine structure: *SHELXTL*; molecular graphics: *SHELXTL*; software used to prepare material for publication: *SHELXTL* and *PLATON* (Spek, 2003 ▶).

## Supplementary Material

Crystal structure: contains datablocks global, I. DOI: 10.1107/S1600536808026305/zl2135sup1.cif

Structure factors: contains datablocks I. DOI: 10.1107/S1600536808026305/zl2135Isup2.hkl

Additional supplementary materials:  crystallographic information; 3D view; checkCIF report

## Figures and Tables

**Table 1:** Selected centroid⋯centroid distances (Å)

*Cg*1⋯*Cg*1^i^	3.7867 (1)
*Cg*2⋯*Cg*3^ii^	4.5626 (3)

Symmetry codes: (i) 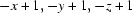; (ii) 
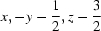
. *Cg*1, *Cg*2, and *Cg*3 are the centroids of the C1–C6, C8–C13 and C15–C20 benzene rings, respectively.

**Table 2:** Hydrogen-bond geometry (Å, °)

*D*—H⋯*A*	*D*—H	H⋯*A*	*D*⋯*A*	*D*—H⋯*A*
O1—H1*O*1⋯N1	0.94	1.83	2.6568 (17)	145
O3—H1*O*3⋯O2^i^	0.96	1.64	2.5919 (18)	171
O4—H1*O*4⋯O3^iii^	0.90	1.87	2.7403 (16)	162
N2—H1*N*2⋯O2	0.88	1.84	2.5954 (18)	143
N2—H1*N*2⋯N1	0.88	2.37	2.7245 (19)	104
C16—H16*A*⋯O1^iv^	0.95	2.55	3.439 (2)	157
C17—H17*A*⋯O4^v^	0.95	2.51	3.381 (2)	152
C11—H11*A*⋯*Cg*4^vi^	0.95	2.97	3.619 (2)	126

Symmetry codes: (i) 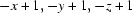; (iii) 
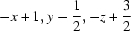
; (iv) 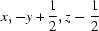; (v) 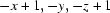; (vi) 
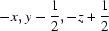
. *Cg*4 is the centroid of the C21–C26 benzene ring.

**Fig. 1.**
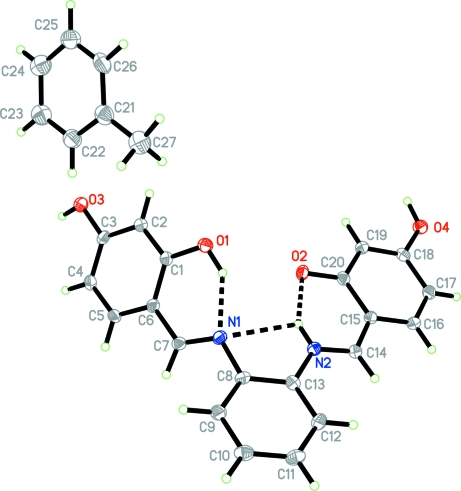
The asymmetric unit of (I), showing 50% probability displacement ellipsoids and the atomic numbering. Intramolecular hydrogen bonds are drawn as dashed lines.

**Fig. 2.**
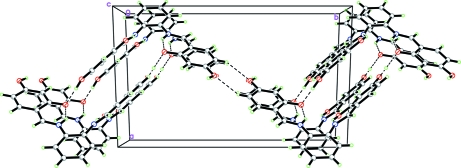
The crystal packing of (I), viewed down the c axis, showing the molecular chains along the b axis and stacking of these chains along the c-axis. Hydrogen bonds are drawn as dashed lines. The toluene molecules were omitted for clarity.

**Fig. 3.**
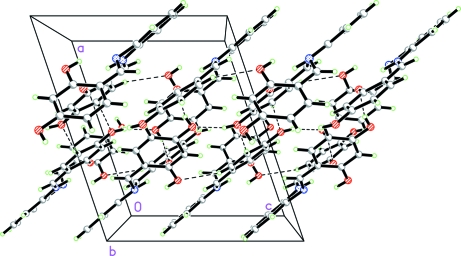
The crystal packing of (I), showing 1-D extended chains along the c axis. The toluene molecules were omitted for clarity.

**Fig. 4.**
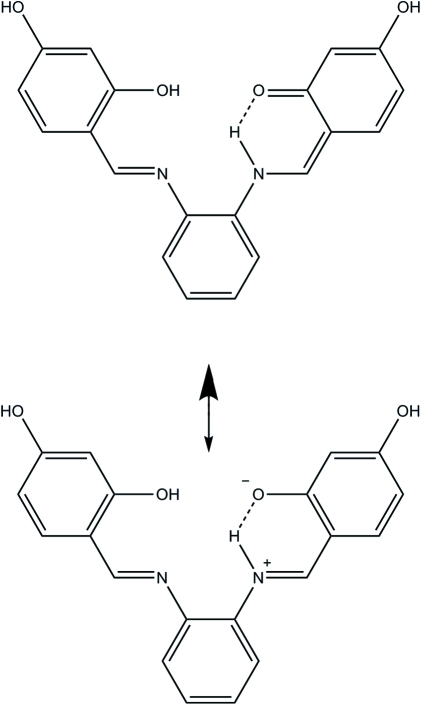
The charge neutral β-keto amine (main component) form and the valence tautomer via partial delocalization of electron pairs along the N—C—C—C—O atom chain (small contribution) in the title compound.

## supplementary crystallographic information

### Comment

Schiff bases have received much attention because of their potential applications with some of these compounds exhibiting various pharmacological activities, as noted by their anticancer (Dao *et al.*, 2000), anti-HIV (Sriram *et al.*, 2006), antibacterial and antifungal (Karthikeyan *et al.*, 2006) properties. Although numerous transition-metal complexes of Schiff bases have been structurally characterized (Granovski *et al.*, 1993), relatively few free Schiff bases have been similarly characterized. *N*-substituted salicylaldimines show photochromism and thermochromism in the solid state. These effects are produced by intramolecular proton transfer associated with a change in the π-electron configuration (Hajioudis *et al.* 1987). In addition, some of them may be used as analytical reagents for the determination of trace elements (Eltayeb & Ahmed, 2005*a,b*) such as nickel in some natural food products (Fakhari *et al.*, 2005) or biologically important species (Shahrokhian *et al.*, 2000). As part of a general study of tetradenate and bidentate Schiff bases (Fun, Kargar & Kia 2008; Fun, Kia & Kargar 2008; Fun, Mirkhani *et al.*, 2008*a,b*), we determined the structure of the title compound.

The title compound was synthesized from *o*-phenylenediamine by reaction with two equivalents of 2,4-dihydroxybenzaldehyde, and the expected reaction product would thus have been the bis-Schiff base 4-((*E*)-(2-((*E*)-2-hydroxybenzylideneamino)phenylimino)methyl) benzene-1,3-diol. The actual molecule obtained in the solid state is however its prototropic tautomer formed by transfer of one of the ortho-hydroxyl protons onto the adjacent imine unit. The proton transfer is accompanied by a shift of electron pairs as is evident from the observed C20–O2 and C14–N2 bond distances of 1.305 (2) and 1.315 (2) Å, which are consistent with C═O and C—N distances (Allen *et al.*, 1987), respectively. The formation of a C═O keto group rather than a C-O^-^ phenolate is also obvious by comparison with the other three phenol C-OH groups in the structure, which are about 0.05 Å longer than C20—O2. The actual molecule present in the solid state is thus the charge neutral β-keto amine (Z)-3-hydroxy-6-((2-((E)-2- hydroxybenzylideneamino)phenylamino)methylene)cyclohexa-2,4-dienone (top isomer in Fig. 4), with a small contribution of its zwitter-ionic valence tautomer via partial delocalization of electron pairs along the atom chain N2—C14—C15—C20—O2 (bottom tautomer in Fig. 4). The other imine group did not undergo proton transfer and is present in its original Shiff base state. Both the imine as well as the amine units are stabilized by strong O—H···N and N—H···O hydrogen bonds (Table 2) that generate *S*(6) ring motifs whereas the intramolecular N—H···N hydrogen bond between the amine and imine (Table 2) exhibits an *S*(5) ring motif (Bernstein *et al.*, 1995). Bond lengths and angles are in normal ranges (Allen *et al.*, 1987) and comparable to those in related structures (Eltayeb *et al.*, 2007*a,b*; Cakir *et al.* 2002; Karabiyik *et al.*, 2007). The C8–C13 phenyl ring makes a dihedral angle of 51.99 (8)° with the dihydroxyphenyl ring (C1–C6/O1/O3) and 12.95 (9)° with the keto-hydroxyphenyl ring (C15–C20/O2/O4). In the crystal packing (Fig. 2), additional O—H···O hydrogen bonds and weak C—H···O interactions (Table 2) link the molecules into one dimensional zigzag extended chains along the *b* axis and these chains are further stacked (Fig. 2 & 3) along the *c* axis thus forming two-dimensional extended networks parallel to the *bc* plane. The crystal is further stabilized by weak C—H···π interactions (Table 2). The short distance between the centroids of the six-membered rings prove an existence of π···π interactions (Table 1).

### Experimental

The title compound was synthesized by adding 2,4-dihydroxybenzaldehyde (0.552 g, 4 mmol) to a solution of *o*-phenylenediamine (0.216 g, 2 mmol) in ethanol (20 ml). The mixture was refluxed with stirring for half an hour. The resultant yellow solution was filtered. Yellow single crystals of the title compound suitable for *X*-ray structure determination were recrystallized from a mixture of THF/toluene (2/1) by slow evaporation of the solvents at room temperature over several days.

### Refinement

Hydroxyl and amine/imine H atoms were located from the difference Fourier map and refined as riding on the parent atoms with isotropic refinement of the displacement parameters. The remaining H atoms were geometrically located and refined as riding model. A rotating group model was used for the methyl groups.

### Crystal data

**Table:**

C_20_H_16_N_2_O_4_·C_7_H_8_	*F*_000_ = 928
*M**_r_* = 440.48	*D*_x_ = 1.380 Mg m^−^^3^
Monoclinic, *P*2_1_/*c*	Mo *K*α radiation λ = 0.71073 Å
Hall symbol: -P 2ybc	Cell parameters from 5653 reflections
*a* = 11.9753 (3) Å	θ = 2.4–29.6º
*b* = 18.8539 (5) Å	µ = 0.09 mm^−^^1^
*c* = 9.9240 (2) Å	*T* = 100.0 (1) K
β = 108.819 (1)º	Plate, yellow
*V* = 2120.87 (9) Å^3^	0.25 × 0.13 × 0.02 mm
*Z* = 4	

### Data collection

**Table:**

Bruker SMART APEXII CCD area-detector diffractometer	6233 independent reflections
Radiation source: fine-focus sealed tube	4023 reflections with *I* > 2σ(*I*)
Monochromator: graphite	*R*_int_ = 0.039
*T* = 100.0(1) K	θ_max_ = 30.2º
φ and ω scans	θ_min_ = 2.2º
Absorption correction: multi-scan(*SADABS*; Bruker, 2005)	*h* = −14→16
*T*_min_ = 0.954, *T*_max_ = 0.994	*k* = −20→26
24830 measured reflections	*l* = −13→14

### Refinement

**Table:**

Refinement on *F*^2^	Secondary atom site location: difference Fourier map
Least-squares matrix: full	Hydrogen site location: inferred from neighbouring sites
*R*[*F*^2^ > 2σ(*F*^2^)] = 0.058	H-atom parameters constrained
*wR*(*F*^2^) = 0.163	*w* = 1/[σ^2^(*F*_o_^2^) + (0.0708*P*)^2^ + 0.4015*P*] where *P* = (*F*_o_^2^ + 2*F*_c_^2^)/3
*S* = 1.11	(Δ/σ)_max_ < 0.001
6233 reflections	Δρ_max_ = 0.76 e Å^−^^3^
299 parameters	Δρ_min_ = −0.32 e Å^−^^3^
Primary atom site location: structure-invariant direct methods	Extinction correction: none

### Special details

**Table:**

Experimental. The low-temperature data was collected with the Oxford Cyrosystem Cobra low-temperature attachment.
Geometry. All e.s.d.'s (except the e.s.d. in the dihedral angle between two l.s. planes) are estimated using the full covariance matrix. The cell e.s.d.'s are taken into account individually in the estimation of e.s.d.'s in distances, angles and torsion angles; correlations between e.s.d.'s in cell parameters are only used when they are defined by crystal symmetry. An approximate (isotropic) treatment of cell e.s.d.'s is used for estimating e.s.d.'s involving l.s. planes.
Refinement. Refinement of *F*^2^ against ALL reflections. The weighted *R*-factor *wR* and goodness of fit *S* are based on *F*^2^, conventional *R*-factors *R* are based on *F*, with *F* set to zero for negative *F*^2^. The threshold expression of *F*^2^ > σ(*F*^2^) is used only for calculating *R*-factors(gt) *etc*. and is not relevant to the choice of reflections for refinement. *R*-factors based on *F*^2^ are statistically about twice as large as those based on *F*, and *R*- factors based on ALL data will be even larger.

### Fractional atomic coordinates and isotropic or equivalent isotropic displacement parameters (Å^2^)

**Table:**

	*x*	*y*	*z*	*U*_iso_*/*U*_eq_	
N1	0.18268 (12)	0.42566 (7)	0.14400 (14)	0.0168 (3)	
O3	0.50774 (11)	0.63459 (6)	0.61784 (12)	0.0214 (3)	
H1O3	0.5724	0.6608	0.6044	0.032*	
O1	0.23309 (11)	0.45373 (6)	0.41957 (12)	0.0225 (3)	
H1O1	0.2053	0.4270	0.3349	0.034*	
O4	0.48126 (11)	0.07727 (6)	0.62444 (12)	0.0217 (3)	
H1O4	0.4999	0.0995	0.7096	0.033*	
O2	0.31282 (10)	0.29118 (6)	0.39178 (12)	0.0187 (3)	
N2	0.17826 (12)	0.28136 (7)	0.12862 (14)	0.0170 (3)	
H1N2	0.2152	0.3043	0.2079	0.025*	
C8	0.11363 (14)	0.39346 (9)	0.01527 (17)	0.0165 (3)	
C6	0.31241 (14)	0.51993 (9)	0.26313 (17)	0.0161 (3)	
C15	0.27803 (14)	0.18211 (9)	0.26242 (17)	0.0165 (3)	
C13	0.10818 (14)	0.31889 (9)	0.00863 (17)	0.0164 (3)	
C16	0.30131 (15)	0.10819 (9)	0.26262 (18)	0.0194 (4)	
H16A	0.2684	0.0818	0.1775	0.023*	
C7	0.24537 (14)	0.48061 (9)	0.13868 (17)	0.0165 (3)	
H7A	0.2475	0.4959	0.0483	0.020*	
C9	0.04597 (15)	0.43207 (9)	−0.10182 (17)	0.0193 (4)	
H9A	0.0487	0.4824	−0.0988	0.023*	
C19	0.39752 (15)	0.18616 (9)	0.51268 (17)	0.0177 (3)	
H19A	0.4305	0.2113	0.5994	0.021*	
C5	0.38949 (15)	0.57342 (8)	0.25065 (17)	0.0176 (4)	
H5A	0.3961	0.5834	0.1597	0.021*	
C1	0.30495 (14)	0.50578 (8)	0.39969 (17)	0.0163 (3)	
C17	0.36973 (15)	0.07431 (9)	0.38208 (18)	0.0198 (4)	
H17A	0.3853	0.0250	0.3804	0.024*	
C18	0.41709 (15)	0.11407 (9)	0.50847 (17)	0.0176 (3)	
C2	0.36978 (14)	0.54490 (9)	0.51645 (17)	0.0175 (4)	
H2A	0.3627	0.5358	0.6075	0.021*	
C12	0.03656 (15)	0.28533 (9)	−0.11326 (17)	0.0204 (4)	
H12A	0.0333	0.2350	−0.1173	0.024*	
C20	0.32949 (14)	0.22283 (9)	0.39033 (17)	0.0164 (3)	
C4	0.45602 (15)	0.61205 (9)	0.36648 (17)	0.0185 (4)	
H4A	0.5083	0.6479	0.3559	0.022*	
C14	0.20597 (15)	0.21362 (9)	0.13726 (17)	0.0176 (4)	
H14A	0.1756	0.1849	0.0548	0.021*	
C10	−0.02551 (15)	0.39818 (10)	−0.22315 (18)	0.0225 (4)	
H10A	−0.0714	0.4253	−0.3024	0.027*	
C3	0.44526 (14)	0.59760 (9)	0.49988 (17)	0.0168 (3)	
C11	−0.02984 (15)	0.32460 (10)	−0.22838 (18)	0.0231 (4)	
H11A	−0.0786	0.3013	−0.3114	0.028*	
C23	0.30310 (18)	0.71109 (11)	0.7808 (2)	0.0329 (5)	
H23A	0.3583	0.7384	0.7523	0.040*	
C25	0.2145 (2)	0.67854 (11)	0.9559 (2)	0.0352 (5)	
H25A	0.2093	0.6830	1.0490	0.042*	
C26	0.13983 (19)	0.63297 (10)	0.8595 (2)	0.0314 (5)	
H26A	0.0822	0.6073	0.8867	0.038*	
C22	0.22868 (18)	0.66449 (10)	0.6864 (2)	0.0293 (4)	
H22A	0.2339	0.6604	0.5932	0.035*	
C21	0.14696 (17)	0.62366 (10)	0.7224 (2)	0.0292 (4)	
C24	0.29687 (18)	0.71778 (11)	0.9173 (2)	0.0350 (5)	
H24A	0.3487	0.7490	0.9837	0.042*	
C27	0.0688 (2)	0.57353 (12)	0.6196 (2)	0.0402 (5)	
H27A	0.1166	0.5365	0.5954	0.060*	
H27B	0.0242	0.5992	0.5331	0.060*	
H27C	0.0139	0.5517	0.6622	0.060*	

### Atomic displacement parameters (Å^2^)

**Table:**

	*U*^11^	*U*^22^	*U*^33^	*U*^12^	*U*^13^	*U*^23^
N1	0.0194 (7)	0.0153 (7)	0.0144 (7)	0.0009 (6)	0.0036 (6)	−0.0015 (5)
O3	0.0251 (7)	0.0191 (6)	0.0189 (6)	−0.0058 (5)	0.0056 (5)	−0.0025 (5)
O1	0.0294 (7)	0.0215 (7)	0.0167 (6)	−0.0103 (5)	0.0076 (5)	−0.0025 (5)
O4	0.0273 (7)	0.0183 (6)	0.0174 (6)	0.0051 (5)	0.0043 (5)	0.0029 (5)
O2	0.0224 (6)	0.0131 (6)	0.0187 (6)	−0.0011 (5)	0.0039 (5)	−0.0009 (5)
N2	0.0183 (7)	0.0169 (7)	0.0137 (7)	−0.0029 (6)	0.0025 (6)	−0.0007 (5)
C8	0.0163 (8)	0.0201 (9)	0.0131 (8)	−0.0010 (7)	0.0048 (6)	−0.0024 (6)
C6	0.0180 (8)	0.0144 (8)	0.0150 (8)	0.0017 (6)	0.0040 (6)	−0.0004 (6)
C15	0.0189 (8)	0.0162 (8)	0.0163 (8)	−0.0011 (7)	0.0082 (7)	−0.0006 (6)
C13	0.0152 (8)	0.0170 (8)	0.0172 (8)	0.0002 (6)	0.0053 (7)	0.0015 (6)
C16	0.0261 (9)	0.0173 (9)	0.0168 (8)	−0.0008 (7)	0.0099 (7)	−0.0025 (7)
C7	0.0188 (8)	0.0165 (8)	0.0139 (8)	0.0033 (7)	0.0047 (7)	0.0005 (6)
C9	0.0218 (9)	0.0181 (9)	0.0169 (8)	0.0033 (7)	0.0047 (7)	0.0013 (7)
C19	0.0196 (8)	0.0176 (9)	0.0151 (8)	−0.0014 (7)	0.0044 (7)	−0.0009 (6)
C5	0.0212 (9)	0.0157 (8)	0.0168 (8)	0.0027 (7)	0.0073 (7)	0.0031 (6)
C1	0.0180 (8)	0.0133 (8)	0.0174 (8)	0.0012 (6)	0.0055 (7)	0.0008 (6)
C17	0.0259 (9)	0.0148 (8)	0.0214 (9)	0.0022 (7)	0.0115 (7)	0.0005 (7)
C18	0.0179 (8)	0.0186 (9)	0.0177 (8)	0.0022 (7)	0.0077 (7)	0.0034 (7)
C2	0.0206 (9)	0.0162 (8)	0.0156 (8)	0.0005 (7)	0.0059 (7)	−0.0002 (6)
C12	0.0195 (9)	0.0194 (9)	0.0205 (9)	−0.0045 (7)	0.0041 (7)	−0.0035 (7)
C20	0.0163 (8)	0.0161 (8)	0.0182 (8)	−0.0010 (6)	0.0074 (7)	0.0003 (6)
C4	0.0202 (9)	0.0143 (8)	0.0207 (9)	−0.0003 (7)	0.0062 (7)	0.0011 (7)
C14	0.0214 (9)	0.0156 (8)	0.0167 (8)	−0.0025 (7)	0.0074 (7)	−0.0007 (6)
C10	0.0197 (9)	0.0285 (10)	0.0159 (8)	0.0037 (7)	0.0012 (7)	0.0014 (7)
C3	0.0179 (8)	0.0142 (8)	0.0164 (8)	0.0008 (6)	0.0028 (7)	−0.0034 (6)
C11	0.0197 (9)	0.0291 (10)	0.0168 (9)	−0.0014 (8)	0.0009 (7)	−0.0037 (7)
C23	0.0296 (11)	0.0343 (12)	0.0362 (12)	0.0008 (9)	0.0124 (9)	0.0017 (9)
C25	0.0458 (13)	0.0309 (11)	0.0313 (11)	0.0098 (10)	0.0158 (10)	0.0044 (9)
C26	0.0341 (11)	0.0258 (11)	0.0402 (12)	0.0053 (9)	0.0201 (10)	0.0106 (9)
C22	0.0307 (11)	0.0280 (10)	0.0316 (11)	0.0033 (9)	0.0134 (9)	0.0029 (8)
C21	0.0292 (10)	0.0275 (10)	0.0311 (11)	0.0057 (8)	0.0101 (9)	0.0055 (8)
C24	0.0330 (11)	0.0363 (12)	0.0336 (11)	0.0063 (9)	0.0076 (9)	−0.0001 (9)
C27	0.0429 (13)	0.0380 (13)	0.0415 (13)	−0.0042 (10)	0.0161 (11)	0.0008 (10)

### Geometric parameters (Å, °)

**Table:**

N1—C7	1.290 (2)	C5—H5A	0.9500
N1—C8	1.415 (2)	C1—C2	1.382 (2)
O3—C3	1.3608 (19)	C17—C18	1.413 (2)
O3—H1O3	0.9628	C17—H17A	0.9500
O1—C1	1.3608 (19)	C2—C3	1.388 (2)
O1—H1O1	0.9431	C2—H2A	0.9500
O4—C18	1.3513 (19)	C12—C11	1.378 (2)
O4—H1O4	0.9048	C12—H12A	0.9500
O2—C20	1.3048 (19)	C4—C3	1.398 (2)
N2—C14	1.315 (2)	C4—H4A	0.9500
N2—C13	1.406 (2)	C14—H14A	0.9500
N2—H1N2	0.8816	C10—C11	1.389 (3)
C8—C9	1.389 (2)	C10—H10A	0.9500
C8—C13	1.408 (2)	C11—H11A	0.9500
C6—C5	1.399 (2)	C23—C22	1.380 (3)
C6—C1	1.412 (2)	C23—C24	1.387 (3)
C6—C7	1.442 (2)	C23—H23A	0.9500
C15—C14	1.396 (2)	C25—C26	1.379 (3)
C15—C16	1.421 (2)	C25—C24	1.382 (3)
C15—C20	1.441 (2)	C25—H25A	0.9500
C13—C12	1.389 (2)	C26—C21	1.402 (3)
C16—C17	1.363 (2)	C26—H26A	0.9500
C16—H16A	0.9500	C22—C21	1.380 (3)
C7—H7A	0.9500	C22—H22A	0.9500
C9—C10	1.388 (2)	C21—C27	1.482 (3)
C9—H9A	0.9500	C24—H24A	0.9500
C19—C18	1.382 (2)	C27—H27A	0.9800
C19—C20	1.406 (2)	C27—H27B	0.9800
C19—H19A	0.9500	C27—H27C	0.9800
C5—C4	1.377 (2)		
			
Cg1···Cg1^i^	3.7867 (1)	Cg2···Cg3^ii^	4.5626 (3)
			
C7—N1—C8	119.05 (14)	C11—C12—C13	120.40 (16)
C3—O3—H1O3	112.8	C11—C12—H12A	119.8
C1—O1—H1O1	108.3	C13—C12—H12A	119.8
C18—O4—H1O4	117.3	O2—C20—C19	121.60 (15)
C14—N2—C13	127.78 (14)	O2—C20—C15	120.84 (15)
C14—N2—H1N2	112.0	C19—C20—C15	117.56 (15)
C13—N2—H1N2	120.0	C5—C4—C3	118.85 (15)
C9—C8—C13	118.59 (15)	C5—C4—H4A	120.6
C9—C8—N1	122.94 (15)	C3—C4—H4A	120.6
C13—C8—N1	118.38 (14)	N2—C14—C15	122.80 (15)
C5—C6—C1	117.97 (15)	N2—C14—H14A	118.6
C5—C6—C7	119.71 (14)	C15—C14—H14A	118.6
C1—C6—C7	122.32 (15)	C9—C10—C11	119.84 (16)
C14—C15—C16	118.90 (15)	C9—C10—H10A	120.1
C14—C15—C20	121.53 (15)	C11—C10—H10A	120.1
C16—C15—C20	119.57 (15)	O3—C3—C2	117.60 (14)
C12—C13—N2	122.70 (15)	O3—C3—C4	121.45 (15)
C12—C13—C8	120.12 (15)	C2—C3—C4	120.95 (15)
N2—C13—C8	117.18 (14)	C12—C11—C10	120.08 (16)
C17—C16—C15	121.56 (15)	C12—C11—H11A	120.0
C17—C16—H16A	119.2	C10—C11—H11A	120.0
C15—C16—H16A	119.2	C22—C23—C24	119.6 (2)
N1—C7—C6	123.24 (15)	C22—C23—H23A	120.2
N1—C7—H7A	118.4	C24—C23—H23A	120.2
C6—C7—H7A	118.4	C26—C25—C24	120.16 (19)
C10—C9—C8	120.97 (16)	C26—C25—H25A	119.9
C10—C9—H9A	119.5	C24—C25—H25A	119.9
C8—C9—H9A	119.5	C25—C26—C21	121.50 (19)
C18—C19—C20	120.93 (15)	C25—C26—H26A	119.3
C18—C19—H19A	119.5	C21—C26—H26A	119.3
C20—C19—H19A	119.5	C21—C22—C23	122.41 (19)
C4—C5—C6	121.86 (15)	C21—C22—H22A	118.8
C4—C5—H5A	119.1	C23—C22—H22A	118.8
C6—C5—H5A	119.1	C22—C21—C26	116.88 (19)
O1—C1—C2	118.28 (14)	C22—C21—C27	121.39 (18)
O1—C1—C6	120.95 (14)	C26—C21—C27	121.72 (19)
C2—C1—C6	120.77 (15)	C25—C24—C23	119.4 (2)
C16—C17—C18	118.67 (16)	C25—C24—H24A	120.3
C16—C17—H17A	120.7	C23—C24—H24A	120.3
C18—C17—H17A	120.7	C21—C27—H27A	109.5
O4—C18—C19	122.31 (15)	C21—C27—H27B	109.5
O4—C18—C17	116.02 (15)	H27A—C27—H27B	109.5
C19—C18—C17	121.66 (15)	C21—C27—H27C	109.5
C1—C2—C3	119.59 (15)	H27A—C27—H27C	109.5
C1—C2—H2A	120.2	H27B—C27—H27C	109.5
C3—C2—H2A	120.2		
			
C7—N1—C8—C9	−43.9 (2)	N2—C13—C12—C11	−179.57 (15)
C7—N1—C8—C13	139.83 (16)	C8—C13—C12—C11	−0.2 (2)
C14—N2—C13—C12	14.2 (3)	C18—C19—C20—O2	−178.20 (15)
C14—N2—C13—C8	−165.19 (16)	C18—C19—C20—C15	2.0 (2)
C9—C8—C13—C12	0.2 (2)	C14—C15—C20—O2	−2.0 (2)
N1—C8—C13—C12	176.67 (14)	C16—C15—C20—O2	177.67 (15)
C9—C8—C13—N2	179.64 (14)	C14—C15—C20—C19	177.82 (15)
N1—C8—C13—N2	−3.9 (2)	C16—C15—C20—C19	−2.5 (2)
C14—C15—C16—C17	−179.13 (15)	C6—C5—C4—C3	−0.5 (2)
C20—C15—C16—C17	1.2 (2)	C13—N2—C14—C15	179.10 (15)
C8—N1—C7—C6	175.76 (15)	C16—C15—C14—N2	178.73 (15)
C5—C6—C7—N1	172.13 (15)	C20—C15—C14—N2	−1.6 (2)
C1—C6—C7—N1	−6.8 (3)	C8—C9—C10—C11	−0.1 (3)
C13—C8—C9—C10	−0.1 (2)	C1—C2—C3—O3	−179.73 (14)
N1—C8—C9—C10	−176.39 (15)	C1—C2—C3—C4	0.5 (2)
C1—C6—C5—C4	−0.5 (2)	C5—C4—C3—O3	−179.24 (15)
C7—C6—C5—C4	−179.50 (15)	C5—C4—C3—C2	0.5 (2)
C5—C6—C1—O1	−178.99 (14)	C13—C12—C11—C10	0.0 (3)
C7—C6—C1—O1	0.0 (2)	C9—C10—C11—C12	0.1 (3)
C5—C6—C1—C2	1.6 (2)	C24—C25—C26—C21	1.7 (3)
C7—C6—C1—C2	−179.48 (15)	C24—C23—C22—C21	0.1 (3)
C15—C16—C17—C18	0.7 (2)	C23—C22—C21—C26	1.9 (3)
C20—C19—C18—O4	−179.47 (15)	C23—C22—C21—C27	−179.43 (19)
C20—C19—C18—C17	−0.1 (2)	C25—C26—C21—C22	−2.7 (3)
C16—C17—C18—O4	178.10 (14)	C25—C26—C21—C27	178.57 (19)
C16—C17—C18—C19	−1.3 (2)	C26—C25—C24—C23	0.4 (3)
O1—C1—C2—C3	178.99 (15)	C22—C23—C24—C25	−1.2 (3)
C6—C1—C2—C3	−1.6 (2)		

Symmetry codes: (i) −*x*+1, −*y*+1, −*z*+1; (ii) *x*, −*y*−1/2, *z*−3/2.

### Hydrogen-bond geometry (Å, °)

**Table:**

*D*—H···*A*	*D*—H	H···*A*	*D*···*A*	*D*—H···*A*
O1—H1O1···N1	0.94	1.83	2.6568 (17)	145
O3—H1O3···O2^i^	0.96	1.64	2.5919 (18)	171
O4—H1O4···O3^iii^	0.90	1.87	2.7403 (16)	162
N2—H1N2···O2	0.88	1.84	2.5954 (18)	143
N2—H1N2···N1	0.88	2.37	2.7245 (19)	104
C16—H16A···O1^iv^	0.95	2.55	3.439 (2)	157
C17—H17A···O4^v^	0.95	2.51	3.381 (2)	152
C11—H11A···Cg4^vi^	0.95	2.97	3.619 (2)	126

Symmetry codes: (i) −*x*+1, −*y*+1, −*z*+1; (iii) −*x*+1, *y*−1/2, −*z*+3/2; (iv) *x*, −*y*+1/2, *z*−1/2; (v) −*x*+1, −*y*, −*z*+1; (vi) −*x*, *y*−1/2, −*z*+1/2.
